# Using Human Milk Fortifiers to Improve the Preoperative Nutritional Status of Infants With Non-restricted Ventricular Septal Defect

**DOI:** 10.3389/fped.2022.900012

**Published:** 2022-06-27

**Authors:** Yun-Feng Lin, Wen-Hao Lin, Shi-Hao Lin, Qi-Liang Zhang, Qiang Chen, Yi-Rong Zheng

**Affiliations:** ^1^Department of Neonatology, Fujian Branch of Shanghai Children's Medical Center Affiliated to Shanghai Jiaotong University School of Medicine, Fuzhou, China; ^2^Department of Neonatology, Fujian Children's Hospital, Fuzhou, China; ^3^Department of Neonatology, Fujian Maternity and Child Health Hospital College of Clinical Medicine for Obstetrics and Gynecology and Pediatrics, Fujian Medical University, Fuzhou, China; ^4^Fujian Key Laboratory of Women and Children's Critical Diseases Research, Fujian Maternity and Child Health Hospital, Fuzhou, China; ^5^Department of Cardiac Surgery, Fujian Children's Hospital, Fuzhou, China; ^6^Department of Cardiac Surgery, Fujian Maternity and Child Health Hospital College of Clinical Medicine for Obstetrics and Gynecology and Pediatrics, Fujian Medical University, Fuzhou, China

**Keywords:** human milk fortifier, breastfeeding, infant, congenital heart disease, nutritional status

## Abstract

**Objective:**

To explore the effects of human milk fortifier (HMF) on improving the preoperative nutritional status of infants with non-restricted ventricular septal defect (VSD).

**Methods:**

A prospective randomized controlled study was conducted in a provincial hospital in China. Participants were randomly divided into an intervention group (*n* = 29) and a control group (*n* = 29). HMFs were added proportionally to the infants' feeds in the intervention group based on breastfeeding status, and the infants in the control group received exclusive breastfeeding as needed. The nutritional status of the two groups was compared 1 month after the intervention.

**Results:**

Compared with the control group, the weight, head circumference, height, albumin level, and prealbumin level of the human milk fortifier group were significantly higher 1 month after the intervention (*p* < 0.05). The STRONGkids score of the HMF group was significantly lower than that of the non-HMF group (*p* < 0.05). There was no significant difference in pneumonia, liver insufficiency, feeding intolerance, or jaundice between the two groups.

**Conclusion:**

The addition of HMFs based on the breastfeeding status of infants with non-restricted VSD can improve the preoperative nutritional status and does not increase the incidence of gastrointestinal complications.

**Clinical Trial Registration:**

http://www.chictr.org.cn/index.aspx, identifier: ChiCTR2000041135.

## Introduction

Ventricular septal defect (VSD) is one of the most common congenital heart diseases. Infants with non-restricted VSD usually have symptoms such as shortness of breath, feeding difficulties, severely affected growth and development, moderate to severe pulmonary hypertension, and even cardiac insufficiency. Due to the large size of the defect and a large number of left to right shunts, surgical correction should be performed in infancy for these patients (1–3). These infants usually have poor nutritional status and low weight before surgery (4). Numerous studies have shown that preoperative nutritional status is closely related to surgery prognosis, and preoperative malnutrition is a high-risk factor for poor outcomes after cardiac surgery in infants (5–7). Therefore, improving the preoperative nutritional status of infants with non-restricted VSD is essential for the postoperative recovery of infants.

Human milk is the ideal food for infants, and breastfeeding is known as the most beneficial way of feeding premature infants (8, 9). However, because infants with non-restricted VSD have many left-to-right shunts, poor cardiac function, abnormal gastrointestinal function, and high energy expenditure, exclusive breastfeeding often cannot meet the nutritional needs of these infants, which might lead to slow growth or weight loss (10). At present, HMFs have been widely used in premature infants to promote their growth and development and improve their nutritional status, and many studies have also demonstrated their safety and effectiveness (11–13). However, there is no research on using HMFs to improve the preoperative nutritional status of infants with non-restricted VSD. This study conducted a prospective randomized controlled study to explore the role of HMFs in improving the preoperative nutritional status of infants with non-restricted VSD.

## Methods

The present study was approved by our hospital's ethics committee and adhered to the tenets of the Declaration of Helsinki. Additionally, all the parents of the patients signed consent forms before participating in the study.

### Study Design

This study was a prospective randomized controlled study conducted by a provincial hospital in China. The inclusion criteria were as follows: (1) infants with non-restricted VSD; (2) infants with no congenital malformation associated with other essential organs; and (3) infants with families who were willing to participate in the study and signed informed consent forms. The exclusion criteria were as follows: (1) patients with severe liver and kidney dysfunction; (2) patients with other severe heart structural abnormalities; (3) patients complicated with digestive insufficiency; (4) patients receiving breast milk via nasal tube feedings; (5) patients who underwent emergency surgery when the condition worsened; and (6) infants who were not exclusively breastfed.

From January 2020 to June 2020, a total of 58 infants who were diagnosed with non-restrictive VSD in our hospital after birth and who were followed up in the outpatient clinic 1 month later were enrolled in this study. All the patients were diagnosed in the neonatal period. Based on the patients' clinical symptoms, ages, and weights, the clinician suggested that the patients should first be discharged for observation and then followed up in the outpatient department 1 month later to determine the timing of surgery.

**Figure 1:**
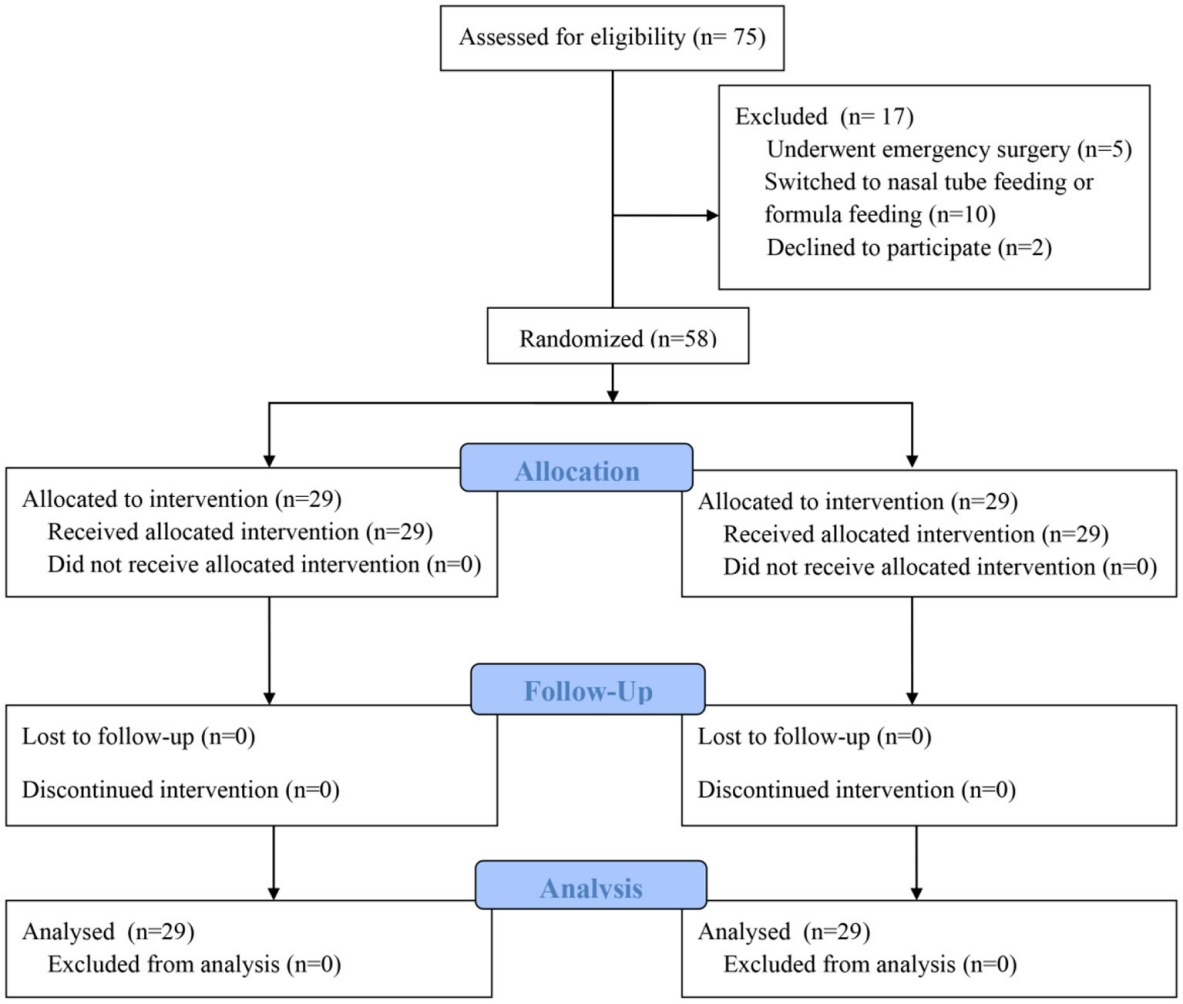
Participants' CONSORT flow diagram.

Calculation of the sample size was based on the difference in the infants' body weights in the two groups in the preliminary experiment. Assuming that the difference between the two independent populations is 10%, with an α = 0.05 and a β = 0.2, the calculated result for each group was 26 participants. Assuming a 10% dropout rate, the total sample size required in this study was 58 patients (29 patients per group). The patients were randomly divided into an intervention group (HMF group) or a control group (non-HMF group) according to computer-generated random numbers. When the patients agreed to participate in this study, the researchers randomized the grouping in a double-blind manner and collected the relevant data. During the same period, 75 patients met the eligibility criteria, and 17 were ultimately excluded, including 5 patients who underwent emergency surgery, 10 patients who were switched to nasal tube feeding or formula feeding during follow-up, and 2 parents of infants who declined to participate (Figure 1).

### Research Tool

The STRONGkids scoring scale was developed by Hulst et al. for nutritional status assessment. It included high-risk diseases, subjective clinical assessment, nutritional intake, and a decrease or no increase in body weight (14). The scoring standard was 2 points for high-risk diseases, 1 point for subjective clinical assessment, 1 point for nutritional intake, and 1 point for a decrease or no increase in body weight. The total score ranged from 0 to 5 points. A score of 0–1 point indicated low nutritional risk, 2–3 points indicated moderate nutritional risk, and 4–5 points indicated severe nutritional risk.

### Intervention Methods

All infants included in the study were fed breast milk. The patients in the intervention group received HMFs proportionally based on breastfeeding status. After the grouping was completed, HMF was added to fortify the patients' nutrition. All patients in the intervention group received breastmilk via bottles. Parents initially prepared more milk with the fortifier and then fed the infant according to their needs, so we could calculate the amount of feed and fortifier. Nestlé, Germany produced the HMF that was used in this study. The HMF was started at a 1/4 dose of fortifier (25 ml human milk: 0.25 g HMF). After excellent tolerance was confirmed for 3 days, the HMF was gradually added to a 1/2 dose of fortifier (25 ml human milk: 0.5 g HMF). After good tolerance was confirmed for another 3 days, the HMF was added to a full dose of fortifier (25 human milk: 1 g HMF). After that, a breast milk fortifier was added at this fixed-rate, and infants were fed on demand (25 ml human milk: 1 g HMF). In the control group, all the patients also received breastmilk via bottles, and the feeding method was the same as that in the intervention group, but with the addition of maltodextrin powder as a placebo.

The nutritional status of the patients was evaluated via outpatient review 1 month after the intervention. If complications occur during the intervention, such as at least one feeding intolerance, necrotizing enterocolitis, pneumonia, liver insufficiency, jaundice, gastrointestinal bleeding, or even death, we stop the intervention and initiate immediate intervention. Nutrition-related indicators, including body weight, head circumference, height, hemoglobin level, albumin level, prealbumin level, and the STRONGkids score, were collected.

### Data Collection and Definitions

The general data, including the patients' age, gender, weight, the diameter of VSD, left ventricle volume, pulmonary artery pressure, and breast milk intake were recorded. The nutritional status and complications between the two groups after the intervention in the two groups of patients were collected by an independent third investigator. Liver insufficiency was defined as ALT > 2 times the normal value of the same age, or total bilirubin >40 mg/L. Feeding intolerance was defined as gastric residual volume >50% of the administered feed volume over the previous 3 h on more than one occasion, the occurrence of any gastric distention and vomiting and/or diarrhea defined as >4 stools per day of a watery consistency. Diagnosis of NEC is carried out by Bell's staging criteria, described in the 1970's, that utilize clinical and radiological characteristics (15).

### Statistical Analysis

SPSS 25.0 software was used for statistical analysis. Continuous data are presented as the mean ± standard deviation and range. The two groups' clinical parameters were compared with the independent samples t-test when these parameters were normally distributed. The χ^2^ test or Fisher's test was used for categorical variables. A *p*-value of < 0.05 was defined as statistically significant.

## Results

The calorie of 100 ml breast milk was 68 kcal, and the calorie of 100 ml breast milk after adding HMF was 85 kcal. Moreover, the contents of protein, fat, and trace elements were also higher in the intervention group (Table 1). There was no statistically significant difference in general information, including weight, head circumference, height, the diameter of the VSD, LV volume z scores, pulmonary artery pressure, age at operation, and breast milk intake between the two groups (Table 2). These results showed that the two groups were homogeneous and comparable.

**Table 1:** The main nutrients of the two milk formulas (per 100 ml).

**Nutrient content**	**Breast milk**	**Breast milk with HMF**
Calories (kcal)	68	85
Protein (g)	1.1	2.5
Fat (g)	3.8	3.8
Calcium (mg)	34	100
Iron (mg)	0	1.4
Phosphorus (mg)	15	59
Vitamin A (IU)	189.8	441
Vitamin B (IU)	2	100
Carbonic acid compound (g)	8	10.3

*HMF, human milk fortifier*.

**Table 2:** Comparison of baseline characteristics of the two groups.

**Characteristics**	**Human milk fortifier group**	**Non-human milk fortifier group**	***P***
Weight (kg)	3.1 ± 0.8	3.0 ± 1.0	0.639
Head circumference (cm)	33.6 ± 1.5	33.4 ± 1.8	0.751
Height (cm)	52.6 ± 3.4	53.4 ± 3.8	0.683
Male/Female	14/15	13/16	0.792
The diameter of VSD (mm)	6.3 ± 1.1	6.6 ± 1.5	0.642
LV volume z scores	2.5 ± 0.4	2.6 ± 0.6	0.891
Pulmonary artery pressure (mmHg)	67.2 ± 10.4	65.6 ± 11.6	0.426
Breast milk intake (ml)	152.7 ± 10.6	153.5 ± 8.5	0.612

*VSD, ventricular septal defect; LV, left ventricle*.

The comparison of nutritional indicators 1 month after the intervention showed that the body weights, head circumferences, heights, albumin levels, and prealbumin levels of the HMF group were significantly higher than those of the non-HMF group (*p* < 0.05). There was no significant difference in hemoglobin levels between the two groups (*p* > 0.05). The STRONGkids score of the HMF group was significantly lower than that of the non-HMF group (*p* < 0.05) (Table 3).

**Table 3:** Comparison of nutritional status between the two groups after the intervention.

**Parameters**	**Human milk fortifier group**	**Non-human milk fortifier group**	***P***
Weight (kg)	4.0 ± 1.1	3.5 ± 1.0	0.035
Head circumference (cm)	37.5 ± 2.0	34.6 ± 2.2	0.041
Height (cm)	59.3 ± 3.2	55.4 ± 4.1	0.043
Albumin (g/L)	39.2 ± 3.6	33.5 ± 5.7	0.025
Prealbumin (mg/L)	231.8 ± 26.1	182.4 ± 31.3	0.011
Hemoglobin (g/L)	123.6 ± 18.4	120.3 ± 16.8	0.453
STRONGkids score	1.8 ± 0.6	3.7 ± 0.9	0.016

The comparison of preoperative complications showed no significant difference in the incidence of pneumonia, liver insufficiency, feeding intolerance, and jaundice between the two groups. There were no complications, such as necrotizing enterocolitis, gastrointestinal bleeding, or death, in the two groups (Table 4). All patients underwent surgical repair of VSDs after the study ended.

**Table 4:** Comparison of complications between the two groups 1 month after the intervention.

**Variable**	**Human milk fortifier group**	**Non-human milk fortifier group**	***P***
Pneumonia	3	4	0.687
Liver insufficiency	1	2	0.553
Feeding intolerance	3	1	0.300
Jaundice	1	2	0.553
Necrotizing enterocolitis	0	0	–
Gastrointestinal bleeding	0	0	–
Death	0	0	–

## Discussion

Non-restricted VSD is a relatively severe type of VSD that often requires surgical treatment in infancy (1–3). With improved surgical techniques, anesthesia, and intensive care, these infants' cure rate has reached more than 95%, but the recovery process is still relatively slow (16, 17). These infants often suffer from feeding difficulties, little or no weight gain, delayed growth and development, and low nutritional status before surgery. The preoperative nutritional status is closely related to the postoperative recovery of the infants. Therefore, to promote infants' postoperative recovery and increase the cure rate, improving the preoperative nutritional status of infants with non-restricted VSDs is vital.

Human milk is the best food for infants, contains many nutrients needed for the growth and development of infants, and has the advantages of enhancing infants' resistance and immunity and protecting the digestive tract mucosa (8, 9). Although human milk has many benefits, many studies have found that infants who are exclusively breastfed may grow at a slower rate than those who are feed formula milk. The content of nutrients, such as protein, calcium, phosphorus, and vitamin D, in human milk is insufficient, so breastfeeding alone cannot meet the high demands of infants for these nutrients (18, 19). Infants with non-restricted VSD have a sizeable left-to-right shunt, which can affect the growth and weight gain of infants and their energy demands. However, due to the limitation of the nutrients and energy provided by human milk, it is impossible to meet these infants' increased energy demands.

HMF contain a variety of proteins, carbohydrates, minerals, and vitamins, which can increase breast milk energy by 5% to 10% and can strengthen the effects of breastfeeding, optimize protein intake and promote development and physical growth. At present, HMFs have been widely used for preterm infants and even very low-weight preterm infants for nutritional support to promote their growth and development and improve their nutritional status, which has achieved good clinical results. Marino LV et al. conducted a retrospective study and showed that HMF could improve the growth and development of low-weight preterm infants who were exclusively breastfed after discharge (20). Tewari VV et al. conducted a prospective case-control study, and the results showed that the application of HMF for premature infants who were exclusively breastfed was beneficial to the growth of weight, head circumference, and height (21). However, there is no research on using HMFs to improve the preoperative nutritional status of infants with non-restricted VSD.

The subjects of this study were infants with non-restricted VSD in outpatient follow-up, and these patients had mild cardiac insufficiency and were given cardiotonic and diuretic drugs. All patients in both groups received breastmilk via bottles, and HMF was applied before surgery. The results of the study showed that the weight, head circumference, albumin levels, and prealbumin levels of the infants in the HMF group were significantly higher than those in the non-HMF group. These results indicated that the HMF could improve nutritional status and reduce malnutrition risk before surgery. Studies have shown that STRONGkids can effectively assess the nutritional risk of children with congenital heart disease during the perioperative period, and provide a basis for nutritional support (22, 23). The STRONGkids scores of infants in the HMF group in this study were significantly lower than those in the non-HMF group, suggesting that HMF can significantly improve the nutritional status of infants with unrestricted ventricular septal defect. Because HMF could increase the osmotic pressure of human milk, some scholars are worried that it might increase gastrointestinal complications, such as necrotizing enterocolitis and feeding intolerance. Numerous studies have shown that the application of HMF for newborns did not increase the incidence of gastrointestinal complications such as necrotizing enterocolitis and feeding intolerance (23, 24). In this study, all the infants were full-term infants. We gradually increased the HMF proportionally. The use of the HMF was started with a 1/4 dose of the fortifier. After observing good tolerance for 3 days, the HMF was added to a 1/2 dose of the fortifier. Then, the HMF was stepped up to a steady dose. After that, a breast milk fortifier was added at this fixed-rate and the infants were fed on demand until the infant came to the clinic. This study also showed no difference in the incidence of necrotizing enterocolitis, gastrointestinal bleeding, feeding intolerance, and jaundice between the two groups, which confirmed that the breast milk booster had acceptable safety and effectiveness.

There were some limitations in this study. This study was a single-center study with small sample size. In this study, non-breastfed or mixed-fed infants were not included, and infants with severe symptoms requiring nasal tube feeding, hospitalization, or emergency surgery, were not also included. These might have caused case selection bias. The limited evaluation indices for the results might affect the accuracy of the results. In addition, the research subjects of this study were limited to unrestricted VSD, and the study period was short. Whether such research results could be generalized remains to be further verified.

## Conclusion

Adding HMFs for breastfeeding infants with non-restricted VSD is beneficial to their overall growth and weight growth, improving preoperative nutritional status, and does not increase the incidence of gastrointestinal complications. It is worthy of clinical application.

## Data Availability Statement

The raw data supporting the conclusions of this article will be made available by the authors, without undue reservation.

## Ethics Statement

The studies involving human participants were reviewed and approved by Fujian Maternity and Child Health Hospital. Written informed consent to participate in this study was provided by the participants' legal guardian/next of kin.

## Author Contributions

Y-FL, QC, and Y-RZ designed the study, performed the statistical analysis, participated in the operation, and drafted the manuscript. W-HL, S-HL, and Q-LZ collected the clinical data. All authors read and approved the final manuscript.

## Funding

This study support by promotion of appropriate technology projects to Rural and Urban Communities, Fujian Province, China (2020TG007).

## Conflict of Interest

The authors declare that the research was conducted in the absence of any commercial or financial relationships that could be construed as a potential conflict of interest.

## Publisher's Note

All claims expressed in this article are solely those of the authors and do not necessarily represent those of their affiliated organizations, or those of the publisher, the editors and the reviewers. Any product that may be evaluated in this article, or claim that may be made by its manufacturer, is not guaranteed or endorsed by the publisher.
