# Supplementary material for: Using Human Milk Fortifiers to Improve the Preoperative Nutritional Status of Infants With Non-restricted Ventricular Septal Defect
Source: Front Pediatr. 2022 Jun 27;10:900012. doi: 10.3389/fped.2022.900012 (PMC9271765; doi:10.3389/fped.2022.900012)
Supplement: Supplementary file 1 [file Table_1.DOCX]

**Table 1. STRONGkids scoring system**

| **Items** | **Explanations** | **Points** |
| --- | --- | --- |
| Subjective clinical assessment | Is the patient in a poor nutritional status judged with subjective clinical assessment: loss of subcutaneous fat and/or loss of muscle mass and/or hollow face? | 1 |
| High risk disease | Is there an underlying illness with risk for malnutrition (see list) or expected major surgery? | 2 |
| Diminished nutritional intake | Is one of the following items present? ▪ Excessive diarrhea (≥5 times/day) and/ or vomiting (>3  times/day) during the last 1–3 days ▪ Reduced food intake during the last 1–3 days ▪ Pre-existing nutritional intervention (e.g., ONS or tube  feeding) ▪ Inadequate nutritional intake because of pain | 1 |
| Weight loss | Is there a weight loss (all ages) and/or no increase in weight/height (infants <1 year) during the last few week–months? | 1 |
